# Flame Retardancy, Fire Behavior, and Flame Retardant Mechanism of Intumescent Flame Retardant EPDM Containing Ammonium Polyphosphate/Pentaerythrotol and Expandable Graphite

**DOI:** 10.3390/ma12244035

**Published:** 2019-12-04

**Authors:** Junsheng Wang, Lei Xue, Bi Zhao, Guide Lin, Xing Jin, Dan Liu, Haibo Zhu, Jinjun Yang, Ke Shang

**Affiliations:** 1 Tianjin Fire Research Institute of Ministry of Emergency Management, 110 Weijin Nan Road, Nankai District, Tianjin 300381, China; wangjunsheng@tfri.com.cn (J.W.); zhaobi@tfri.com.cn (B.Z.); lin64350@163.com (G.L.); jinxing@tfri.com.cn (X.J.); liudan@tfri.com.cn (D.L.); 2 School of Environmental Science and Safety Engineering, Tianjin University of Technology, 391 Binshui Xidao, Xiqing District, Tianjin 300384, China; 18435154189@163.com (L.X.); zhb1506414408@163.com (H.Z.)

**Keywords:** ethylene–propylene–diene rubber, flame retardancy, fire behavior, synergistic flame retardant effect, flame retardant mechanism

## Abstract

The intumescent flame retardant ethylene–propylene–diene rubber (EPDM) was prepared using intumescent flame retardant (IFR), including ammonium polyphosphate (APP) /pentaerythrotol (PER) and expandable graphite (EG), as the flame retardant agent. The effects of IFR and EG on the flame retardancy, fire behavior, and thermal stability of the EPDM were investigated. The results show that IFR and EG have excellent synergistic flame retardant effects. When the mass ratio of IFR to EG is 3:1 and the total addition content is 40 phr, the limiting oxygen index (LOI) value of the EPDM material (EPDM/IFR/EG) can reach 30.4%, and it can pass a V-0 rating in the vertical combustion (UL-94) test. Meanwhile, during the cone calorimetry test, the heat release rate and total heat release of EPDM/IFR/EG are 69.0% and 33.3% lower than that of the pure EPDM, respectively, and the smoke release of the material also decreases significantly, suggesting that the sample shows good fire safety. In addition, the flame retardant mechanism of IFR and EG is systematically investigated by thermogravimetric analysis/infrared spectrometry (TG-IR), Fourier transform infrared spectroscopy (FTIR), X-ray photoelectron spectroscopy (XPS), and scanning electron microscopy (SEM), and the results indicate that IFR and EG have only physical interaction. Moreover, the reason why IFR exhibits a poor flame retardant effect in EPDM materials is explained.

## 1. Introduction

Ethylene–propylene–diene rubber (EPDM), as one of the most useful synthetic rubbers composed of ethylene, propylene, and unsaturated diene, is resistant to heat, light, ozone, and ultraviolet radiation and has been widely used in heat-resistant conveyor belts, cables, wires, gaskets, door and window seals, and so on. However, EPDM is a flammable material with a very low limiting oxygen index (LOI) value, which greatly limits its further development and application [1]. Therefore, it is necessary to improve the flame retardant performance of EPDM [2,3,4].

The halogenated flame retardant [5,6,7], such as decabromodiphenyl oxide and decabromodiphenyl ethane, has a good flame retardant effect on EPDM, which can significantly improve the LOI of EPDM materials used in incorporation with antimony trioxide compounds. However, due to the toxicity and bioaccumulation of halogenated flame retardant causing potential harm to the environment, it is being gradually phased out by the market and replaced by halogen-free flame retardants, particularly metallic hydroxide flame retardants, such as aluminum hydroxide, magnesium hydroxide, hydrotalcite, hydroxyl aluminum oxalate, and so on [8,9,10,11,12,13]. Metallic hydroxide has been extensively used as halogen-free flame retardant in polymeric materials due to its higher decomposition temperature and lower smoke and toxic gas production [14,15], but because of its low flame retardant efficiency, rather a high loading of flame retardant is required to achieve the necessary flame retardant effect, which will deteriorate the overall performances of the composites dramatically.

The intumescent system used for flame retardant materials has been studied initially by Camino [16]. It is an important material for the preparation of halogen-free environmentally friendly flame retardant materials, which are mainly divided into chemical intumescent flame retardant and physical intumescent flame retardant. The former is a flame retardant with carbon, nitrogen, and phosphorus elements, which is mainly composed of an acid source, gas source, and carbon source, which has the advantages of high flame retardant efficiency, low smoke, low toxicity, non-corrosive gas, and low cost [17]. When burning, the chemical intumescent flame retardant can form a uniform carbon foam layer, which has the function of blocking oxygen, heat insulation, and smoke suppression, thence improving the flame retardant property of the polymer. The latter is a kind of flame retardant represented by expandable graphite (EG) [18,19]. Since EG itself cannot form a dense carbon layer, the flame retardancy of it is limited when used alone. Although the efficiency of the intumescent flame retardant is relatively higher compared with metallic hydroxide, it still needs a large amount of addition to achieve the desired flame retardant effect. Therefore, the separate use of the flame retardant often fails to achieve the desired effect. Currently, the research on the preparation of high flame retardant polymer materials by the synergistic flame retardant has become a hot spot in the direction of flame retardant materials research [20,21,22,23,24]. Furthermore, the synergistic flame retardant effect between chemical intumescent flame retardant and EG has been confirmed [25,26]. However, the synergistic flame retardant mechanism of intumescent flame retardant (IFR) and EG in EPDM materials has not been systematically studied. Moreover, according to the literature [27,28,29], IFR exhibits better flame retardant properties in many thermoplastics polymers, such as polypropylene, polyethylene, and ethylene–vinyl acetate copolymer, than in EPDM rubbers, but the cause of this phenomenon has not been studied and discussed so far.

Herein, the typical chemical intumescent flame retardant (IFR, APP/PER) and EG were used as synergistic flame retardants to prepare flame retardant EPDM rubber. The flame retardancy, thermal stability, and combustion behavior were investigated by means of the limiting oxygen index (LOI), vertical burning (UL-94), cone calorimeter (CC), and thermogravimetric analysis (TG). The synergistic flame retardant mechanism of IFR and EG is systematically investigated by thermogravimetric analysis/infrared spectrometry (TG-IR), Fourier transform infrared spectroscopy (FTIR), X-ray photoelectron spectroscopy (XPS), and scanning electron microscopy (SEM). Moreover, the combustion process of the flame retardant EPDM materials was characterized by digital camera and SEM.

## 2. Materials and Methods

### 2.1. Materials

Ethylene–propylene–diene rubber (EPDM, 4640) was supplied by Dow Chemical Company, Michigan, USA. Ammonium polyphosphate (APP, technical grade) was purchased from Yunnan Tianyao Chemical Co. Ltd., Kunming, China. Pentaerythrotol (PER, technical grade) was provided by Tianjin Fengchuan Chemical Reagent Technologies Co. Ltd., Tianjin, China. Melamine (MA, technical grade) was supplied by Tianjin Hongxitai Chemical Trading Co. Ltd., Tianjin, China. Expandable graphite (EG, technical grade) was purchased from Qingdao Tianheda Graphite Co. Ltd., Qingdao, China. Dicumyl peroxide (DCP, chemical pure) was provided by Shanghai Aladdin Bio-Chem Technology Co. Ltd., Shanghai, China. All ingredients were used without further purification.

### 2.2. Preparation of the Flame Retardant EPDM Materials

EPDM, APP, PER, MA, and EG were dried in an oven at 8 °C for 6 h before the preparation of flame retardant EPDM. Then, EPDM was mixed uniformly with the intumescent flame retardant and vulcanizing agent in an HTR-300 twin-roll mill (Guangzhou Hartek Machinery Co. Ltd., Guangzhou, China) at 100 °C. After placement at room temperature for 12 h, the resulting mixture was vulcanized at 160 °C for 15 min under 10 MPa pressure using an R-3212 press vulcanizer (Wuhan Qien Science and Technology Development Co. Ltd., Wuhan, China) and compression-molded into sheets for further analysis.

### 2.3. Characterization and Measurements

The flame retardancy of the flame retardant EPDM was investigated by the limiting oxygen index test, which was tested on an JF-3 oxygen index meter (Nanjing Jiangning Analytical Instrument Co. Ltd., Nanjing, China) according to GB/T 10707-2008, and the dimensions of all samples are 120 mm × 6.5 mm × 3 mm.

The flame retardancy of the flame retardant EPDM was further investigated by the vertical burning test (UL-94), which was performed on a CZF-2 instrument (Nanjing Jiangning Analytical Instrument Co. Ltd., Nanjing, China) according to GB/T 10707-2008, and the dimensions of all samples are 130 mm × 13 mm × 3 mm.

Thermogravimetric analysis (TGA) was carried to detect the thermal stability of the EPDM materials on a STA 6000 thermogravimetric analyzer (PerkinElmer, MA, USA) at a heating rate of 10 °C min^−1^ under N_2_.

The combustion behaviors were measured by a cone calorimeter (CC) device (Fire Testing Technology, West Sussex, UK) according to ISO 5660-1. Samples with a size of 100 mm × 100 mm × 3 mm were tested under a heat flux of 35 kW m^−2^.

Thermogravimetric analysis/infrared spectrometry (TG-IR) was performed using an STA 6000 thermogravimetric analyzer (PerkinElmer, MA, USA) and a Frontier FTIR spectrometer (PerkinElmer, MA, USA) at a heating rate of 10 °C min^−1^ under N_2_ in order to analyze the gaseous substances produced by the decomposition of EPDM materials during the heating process.

To investigate the chemical structures of the carbon residues after cone calorimeter tests, the Fourier transform infrared (FTIR) spectra of the carbon residues were recorded on a Frontier FTIR spectrometer (PerkinElmer, MA, USA) by KBr-pellet.

X-ray photoelectron spectroscopy (XPS) were recorded by a Thermo ESCALAB 250XI (Thermo Fisher Scientific, MA, USA), so as to reveal the status of elements in the carbon residues of the flame retardant EPDM materials after a cone calorimeter test.

Morphological microstructure of the samples was characterized with an SU3500 scanning electron microscopy (SEM; HITACHI, Tokyo, Japan) at acceleration voltage of 15 kV.

## 3. Results

### 3.1. Formulation and LOI, UL-94 Test

LOI and UL-94 test are the most commonly used and effective means of characterizing the flame retardant properties of polymer materials. The former is often used to judge the degree of difficulty for the materials to burn in air, while the latter is commonly used to evaluate the resistance of the material to ignition. The formulation, LOI values, and UL-94 test results of the flame retardant EPDM are summarized in Table 1. The results show that the pure EPDM is a flammable material with a very low LOI value of 17.8 and no UL-94 rating. When the traditional intumescent flame retardant (IFR) is added alone, the flame retardancy of the samples with the addition of APP, PER, and MA is poor, which has a LOI value of 26.4 even with a flame retardant loading of 60 phr. The samples without MA shows better flame retardant property compared with the samples with the addition of MA. A good flame retardancy can be achieved for the samples with an APP and PER ratio of 3:1, which can be attributed to the fact that PER when used as a carbon-forming agent will act as a combustion aid with a high loading, while a good expanded carbon layer can be formed with a proper ratio of APP and PER, thereby improving the flame retardant performances of the samples. Unfortunately, a large amount of flame retardant needs to be added to impart better flame retardant property in the samples, in which the samples with an IFR loading of 40 phr and 60 phr have LOI values of 26.2 and 32.2, respectively. Meanwhile, the flame retardant property of the EPDM is also poor with the addition of EG alone. Although the sample with a low EG loading (30 phr) can reach a V-0 rating in the UL-94 test, the LOI value is only 24.4. Furthermore, the LOI of the sample is only 26.4 even with 50 phr EG.

In order to obtain samples with excellent flame retardant performance, IFR and EG are compounds that are used as synergistic flame retardants, and the LOI and UL-94 test were investigated for the samples with a total flame retardant loading of 40 phr and EG loadings of 5 phr, 10 phr, and 15 phr, respectively. The results show that the sample can achieve excellent flame retardant properties when the feeding mass ratio of IFR and EG is 3:1, which has an LOI value of 30.4 and a V-0 UL-94 rating. Moreover, the flame retardancy of the samples improved as the flame retardant content increased. Overall, the samples have optimal flame retardancy when the feeding mass ratio of IFR and EG is 3:1 and the total flame retardant loading is 40 phr. Thence, four samples are used in the following test, including the virgin EPDM named EPDM, the sample with an IFR loading of 40 phr named EPDM/IFR, the sample with an EG loading of 40 phr named EPDM/EG, and the sample with an IFR and EG loading of 30 phr and 10 phr, respectively, in which the ratio of APP and PER is 3:1, so as to further verify the synergistic flame retardant effect of IFR and EG.

### 3.2. Thermal Stability

**Figure 1:**
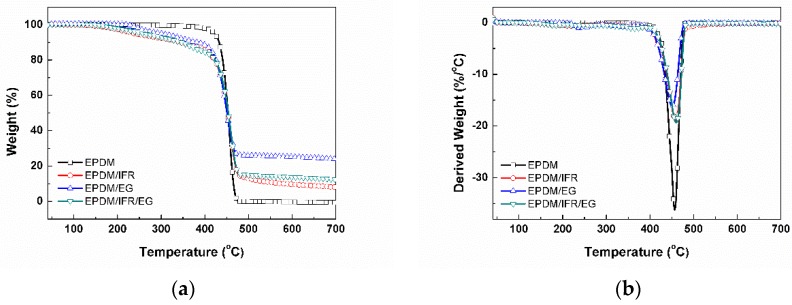
(**a**) Thermogravimetric analysis (TGA) weight loss and (**b**) the corresponding derivative thermogravimetric analysis(DTG) curves of the EPDM materials.

Figure 1 shows the thermogravimetric analysis (TGA) results of the EPDM materials, and the resultant characteristic thermal degradation data are summarized in Table 2, including the onset thermal decomposition temperature (*T*_onset_), the maximum thermal decomposition temperature (*T*_max_), the thermal decomposition rate at the *T*_max_ (d*W*/d*T*), and the residual weight. The pure EPDM has only one thermal decomposition stage at about 400–500 °C corresponding to the thermal decomposition of EPDM. Due to the early decomposition of the intumescent flame retardant, the *T*_onset_ of the EPDM materials with the addition of IFR and EG is reduced. The *T*_max_ of EPDM/EG is also decreased, as the rapid expansion of EG makes the substrate more susceptible to exposure in heat sources. As the dense carbon layer formed by IFR can protect the substrate from thermal decomposition, the *T*_max_ of EPDM/IFR and EPDM/IFR/EG is increased by 2.0 °C and 3.4 °C, respectively, compared with the virgin EPDM, demonstrating an improvement of thermal stability in high temperature regions. Moreover, the good char-forming performance of the flame retardant can protect the substrate from decomposing too fast, so the decomposition rate at the *T*_max_ of the materials also decreased significantly. In addition, EG only releases a certain amount of gas at high temperature, and the graphite component will not decompose; meanwhile, IFR also has good char-forming ability. Therefore, the carbon residue of the EPDM materials with the addition of an intumescent flame retardant is significantly improved, which is 8.0%, 24.0%, and 12.4% for EPDM/IFR, EPDM/EG, and EPDM/IFR/EG, respectively, compared with complete decomposition for the pure EPDM rubber. In summary, although the *T*_onset_ is decreased, the thermal stability of the EPDM materials in high-temperature regions is improved with the addition of IFR and EG.

### 3.3. Combustion Behavior

**Figure 2:**
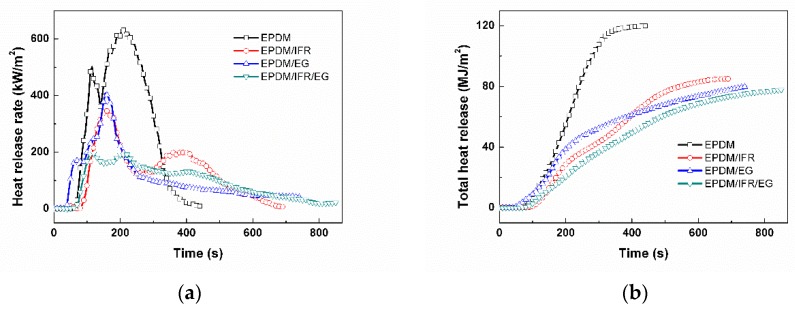
(**a**) Heat release rate (HRR) and (**b**) total heat release (THR) plots of the EPDM materials under a heat flux of 35 kW/m^2^.

**Figure 3:**
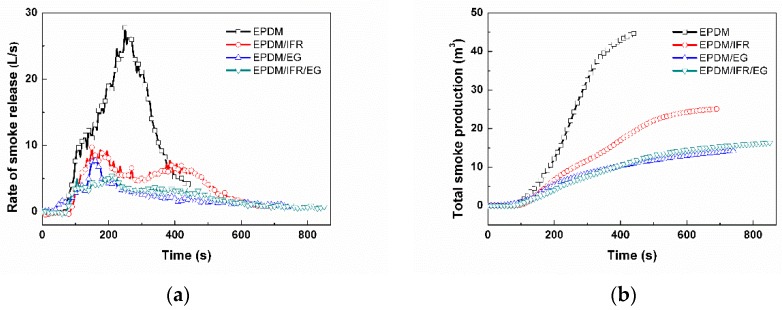
(**a**) Rate of smoke release (RSR) and (**b**) total smoke production (TSP) plots of the EPDM rubbers under a heat flux of 35 kW/m^2^.

The combustion behavior of the flame retardant EPDM materials were further investigated by cone calorimetry. Cone calorimetry is designed according to the principle of oxygen consumption, which is currently recognized as the most ideal small-scale combustion performance test method. The evolution of heat release and smoke release with time are exhibited in Figure 2 and Figure 3, respectively, and the corresponding key parameters are listed in Table 3, including time to ignition (TTI), peak of heat release rate (PHRR), total heat release (THR), time to PHRR (TTPHRR), fire growth rate (FIGRA), total smoke production (TSP), and residue.

The value of TTI implies that combustible gas has come out from the degradation of the polymer and accumulated to a certain concentration [30]. Therefore, a longer TTI value indicates a slower flame spread rate and less degradation of the material. According to the TG data, the thermal decomposition temperatures of IFR and EG are lower than that of EPDM. During the ignition process, EPDM/EG will expand rapidly due to the expansion of EG, resulting in an increased contact area of the material with the thermal radiation source, so the TTI value is much lower than that of the pure EPDM, as shown in Table 3. This result indicates that it is not conducive to the ignition safety of the material with the addition of EG alone. In contrast, a positive impact on the ignition time can be achieved in the case of adding IFR. That’s because IFR will release a large amount of inert gas and thus dilute the combustible gas. Meanwhile, the samples with the addition of IFR have no obvious expansion during ignition, and the expansion of the sample with the addition of IFR and EG together is still much lower than that of EPDM/EG. Consequently, the TTI value of EPDM/IFR and EPDM/IFR/EG extends for 26 s and 18 s, respectively, compared with pure EPDM rubber, indicating an improvement of ignition safety with the addition of IFR.

The HRR, PHRR, and THR are the most important parameters for evaluating the fire safety of the material, in which a lower value indicates a better flame retardant performance. The PHRR and THR of pure EPDM are 637.3 kW/m^2^ and 116.4 MJ/m^2^, respectively, indicating that EPDM is a material with very poor fire safety. The flame retardancy of the EPDM rubber was improved with the addition of IFR and EG. The PHRR of EPDM/IFR and EPDM/EG decrease remarkably by 345.8 kW/m^2^ and 408.0 kW/m^2^, which are respectively 45.7% and 36.0% lower than that of the pure EPDM rubber. When IFR and EG are used together, the PHRR of the EPDM/IFR/EG is further reduced, which is 69.0% lower than that of the pure EPDM, indicating that IFR and EG have a wonderful flame retardant effect on EPDM. The flame retardancy of the samples with the addition of different flame retardant can also be observed through the trend of the HRR curves. The HRR curve of the virgin EPDM has two peaks, which may be because a carbon layer can be formed after burning of the upper substrate, and the carbon layer will be destroyed under the continuous impact of the heat source; thereby, the underlying substrate will continue to burn, resulting in a second HRR peak. The HRR curve of the EPDM/IFR is similar to that of the pure EPDM, which also has two peaks, but the second peak of it is much smaller than that of the pure EPDM. As EG will only form a loose carbon layer and the TTI is short, the substrate will burn completely quickly, resulting in a sharp peak in the HRR curve for the EPDM/EG. The HRR curve of the sample with the addition of IFR and EG together is different from the others. During the combustion process, the burning tendency is relatively gentle for EPDM/IFR/EG, and the HRR peak is very wide, indicating that the carbon layer formed by IFR and EG can well suppress the flame propagation and make the sample not burnt violently. Compared with EPDM/IFR and EPDM/EG, the PHRR values of EPDM/IFR/EG decrease by 43% and 51%. These results demonstrate that IFR and EG have an excellent synergistic flame retardant effect. Meanwhile, the THR values of the samples also correspondingly decrease significantly. The THR values of EPDM/IFR, EPDM/EG, and EPDM/IFR/EG at the end of the burning test decrease by 27.1%, 31.3%, and 33.3%, respectively, compared with the pure EPDM, demonstrating an excellent flame retardant property of the composite materials and a good synergistic flame retardant effect between IFR and EG.

FIGRA is also a very important evaluation parameter for fire safety of materials, which is defined as the ratio of PHRR and TTPHRR. The FIGRA of the materials presents the order of EPDM > EPDM/EG > EPDM/IFR > EPDM/IFR/EG, indicating that the fire safety of the samples is improved significantly with the addition of intumescent flame retardant, especially for EPDM/IFR/EG. Moreover, since the intumescent flame retardant forms a good carbon layer during combustion, the underlying substrate is protected from violent thermal decomposition and burning. The carbon residue of the EPDM materials is enhanced obviously with the addition of intumescent flame retardant, which is 2.9%, 21.3%, 25.7%, and 43.9% for EPDM, EPDM/IFR, EPDM/EG, and EPDM/IFR/EG, respectively.

In most cases, the smoke released in a fire is an important factor that directly causes harm to people, which can cause suffocating or poison, even death. Therefore, it’s particularly necessary to reduce the smoking release of a material. The peak of the rate of smoke release (RSR) and TSP of EPDM are 27.7 L/s and 38.6 m^3^, respectively, indicating that EPDM is a dangerous material that will release a lot of smoke quickly during combustion. The tendency of the rate of smoke release (RSR) curves is similar to that of the HRR curves. The peak of the samples in the RSR curves are greatly reduced with addition of the intumescent flame retardant, especially for EPDM/IFR/EG. Meanwhile, the TSP values of the samples are also reduced significantly, in which the TSP of EPDM/EG, EPDM/IFR, and EPDM/IFR/EG decreased by 35.0%, 63.2%, and 57.8%, respectively, compared with the pure EPDM, indicating an excellent smoke suppression effect of the intumescent flame retardant.

The fire retardancy features of the flame retardant EPDM materials can be compared more intuitively through the ‘Flame Retardancy Index’ (FRI) according to the literature [31]. The “Poor”, “Good”, and “Excellent” flame retardancy performances can be explicitly defined by the FRI values. The FRI values of the EPDM/IFR, EPDM/EG, and EPDM/IFR/EG materials are 3.79, 1.53, and 6.52, respectively, which can all be defined as ‘Good’ flame retardancy performances. Furthermore, the EPDM materials with the addition of IFR and EG together have the best flame retardancy performance according to the FRI values, indicating again that IFR and EG have an excellent synergistic flame retardant effect in EPDM materials.

### 3.4. Gaseous Phase Decomposition Behavior

**Figure 4:**
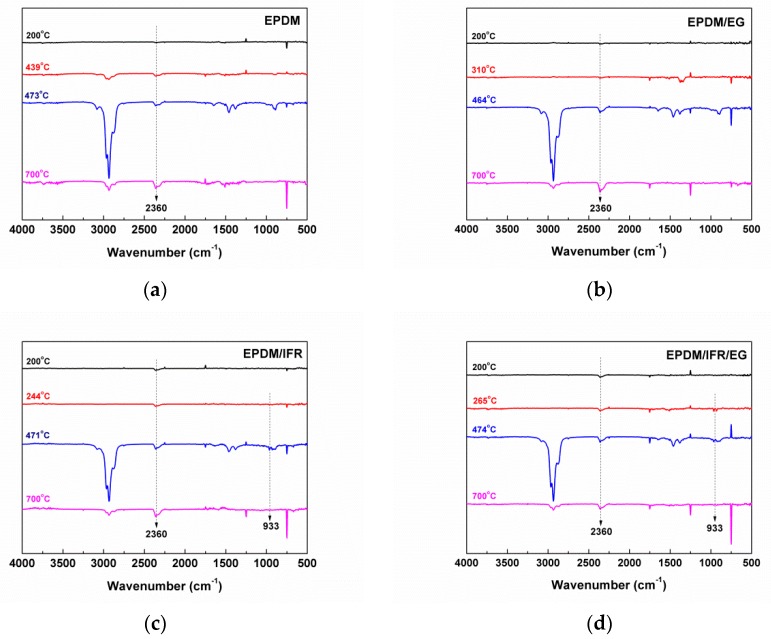
Fourier transform infrared spectroscopy (FTIR) spectra of volatile pyrolysis products for (**a**) EPDM, (**b**) EPDM/EG, (**c**) EPDM/IFR and (**d**) EPDM/IFR/EG at different temperatures. IFR: intumescent flame retardant.

The thermogravimetric analysis-infrared spectrometry (TG-IR) technique is used to analyze gaseous products during the thermal decomposition process, which can contribute to better understanding the degradation mechanisms of polymeric materials. Figure 4 shows the IR spectra of EPDM, EPDM/EG, EPDM/IFR, and EPDM/IFR/EG obtained at 200 °C, *T*_onset_, *T*_max_, and 700 °C. The results show that almost no absorption peaks appear at 200 °C and 700 °C, indicating that the EPDM materials are basically not decomposed at 200 °C and completely decomposed at 700 °C. The main gaseous products of EPDM are CO_2_ (2360 cm^−1^) and hydrocarbon (2800–3100 cm^−1^ and 1300–1600 cm^−1^) [32]. Since EG only releases some inert gas, the main gaseous products of EPDM/EG are similar to those of EPDM. For EPDM/IFR and EPDM/IFR/EG, in addition to the absorption peaks generated by the decomposition of EPDM, a new absorption peak emerges at 933 cm^−1^ (NH_3_), which contributes to the thermal degradation of APP. These inert gases can dilute the combustible gas, thereby playing a flame retardant effect.

### 3.5. Charring Behavior Analysis

**Figure 5:**
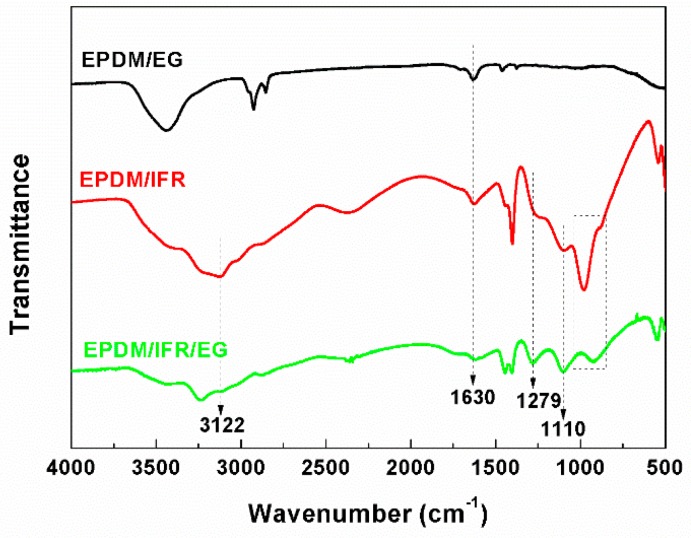
FTIR spectra of char residues for the EPDM materials after the cone calorimeter test.

In order to better understand the solid phase flame retardant mechanism of the flame retardant EPDM materials, the chemical structures of the carbon residues after cone calorimeter tests are investigated by FTIR, and the FTIR spectra are shown in Figure 5. For the carbon residue of EPDM/EG, the absorption peak at 1630 cm^−1^ is assigned to the stretching vibration of the C=C bond, which mainly belongs to EG and the incompletely burned substrate. After the addition of IFR, new peaks at 950–960 cm^−1^, 1110 cm^−1^, 1279 cm^−1^, and 3122 cm^−1^ appear for EPDM/IFR and EPDM/IFR/EG, which is assigned to P–O–P, P–O–C, P=O, and –NH_3_^+^, respectively. These characteristic peaks indicate that APP and PER can promote the formation of a crosslinked carbon layer for the EPDM materials, thereby increasing the content and density of the carbon layer. Moreover, no new chemical bonds form as shown in the spectra, which demonstrate that no chemical interaction occurs between IFR and EG during combustion. However, the peak of P–O–C for EPDM/IFR/EG is relatively higher than EPDM/IFR, indicating that the physical interaction between IFR and EG also helps to promote the formation of the crosslinked carbon layer.

**Figure 6:**
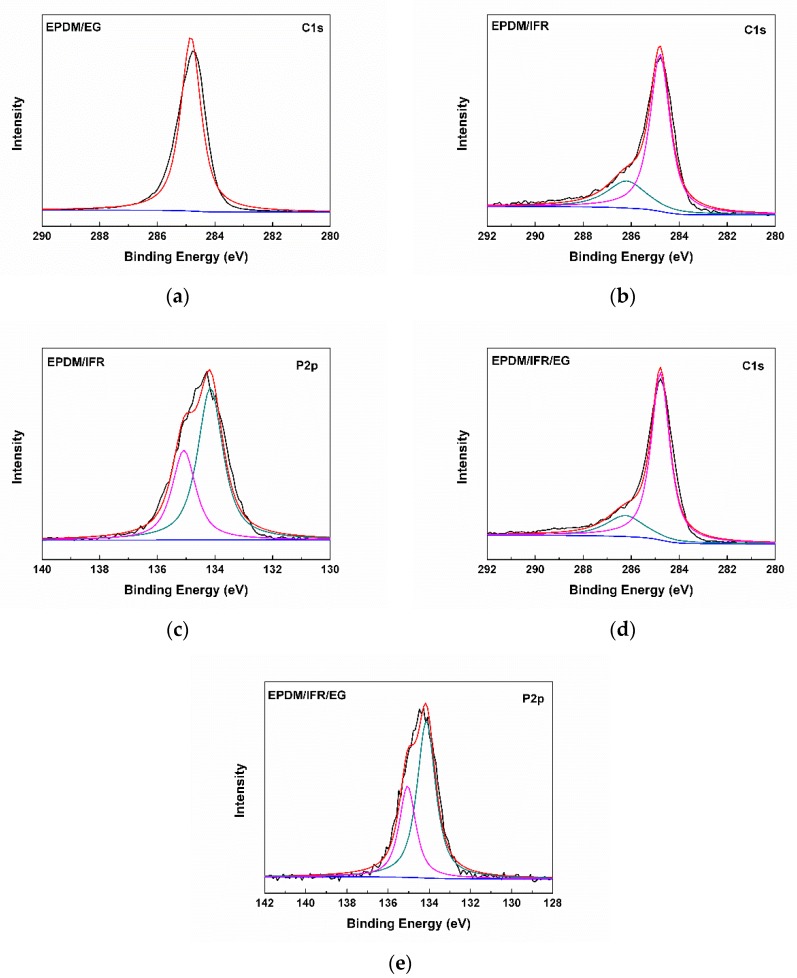
(**a**) X-ray photoelectron spectroscopy (XPS) C_1s_ narrow scan spectrum of the carbon residue of EPDM/EG; (**b**) XPS C_1s_ and (**c**) XPS P_2p_ narrow scan spectrum of the carbon residue of EPDM/IFR; (**d**) XPS C_1s_ and (**e**) XPS P_2p_ narrow scan spectrum of the carbon residue of EPDM/IFR/EG.

To further reveal the status of elements, the carbon residues of the flame retardant EPDM materials after a cone calorimeter test are studied by X-ray photoelectron spectroscopy (XPS), and the XPS C_1s_ and P_2p_ narrow scan spectrum are shown in Figure 6; the corresponding element composition of the carbon residues are summarized in Table 4. According to the literature [33], for the XPS C_1s_ narrow scan spectrum of EPDM/EG, only one peak appears at 284.9 eV, which is attributed to C=C, mainly belonging to EG and the incompletely burned substrate. For the XPS C_1s_ narrow scan spectrum of EPDM/IFR and EPDM/IFR/EG, the peak at 284.8 eV is attributed to C=C and C–C, and the peak at 286.2 eV is assigned to P–O–C and/or C–N. The peaks of the XPS P_2p_ narrow scan spectrum at 134.2 eV and 135.1 eV correspond to P–O and P=O, which may be derived from polyphosphate compounds. The results of the element composition show that the carbon residue of EPDM/EG mainly contains the C element, and a small amount of O element may be derived from vulcanizing agents and water. Compared with EPDM/IFR, the carbon residue of EPDM/IFR/EG has a higher content of the C element and a lower content of O, N, and P elements, as part of the IFR is replaced by EG. Moreover, based on the element composition of EPDM/EG and EPDM/IFR, the calculated values of the C, O, N, and P elements for EPDM/IFR/EG are 49.88%, 43.32%, 4.59%, and 8.82%, respectively. The actual elemental content of O, N, and P are 12.52%, 1.15%, and 2.76% lower than that of the calculated values, while it has a higher content of the C element, demonstrating that IFR and EG have a good synergistic flame retardant effect and can protect the substrate from burning. The overall results also indicate that IFR and EG have an only physical interaction as no new chemical bonds form, which is consistent with the results of FTIR.

**Figure 7:**
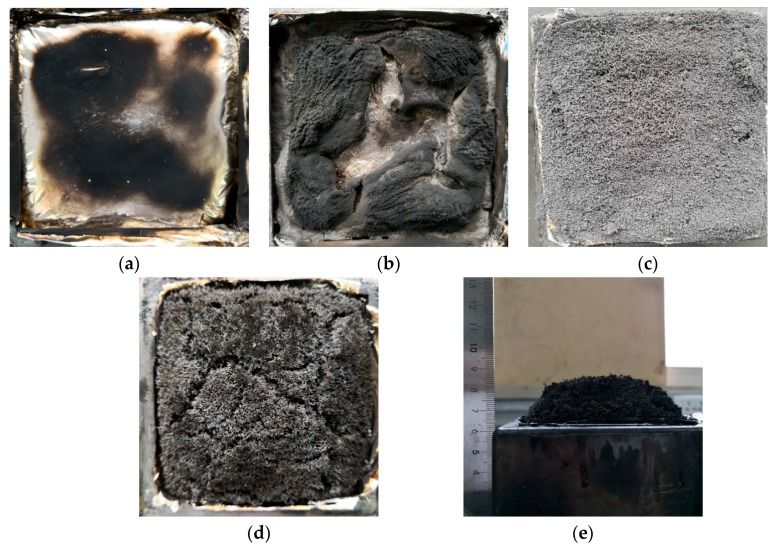
Digital photographs of carbon residues for (**a**) EPDM, (**b**) EPDM/IFR, (**c**) EPDM/EG, and (**d**,**e**) EPDM/IFR/EG after the cone calorimeter test.

**Figure 8:**
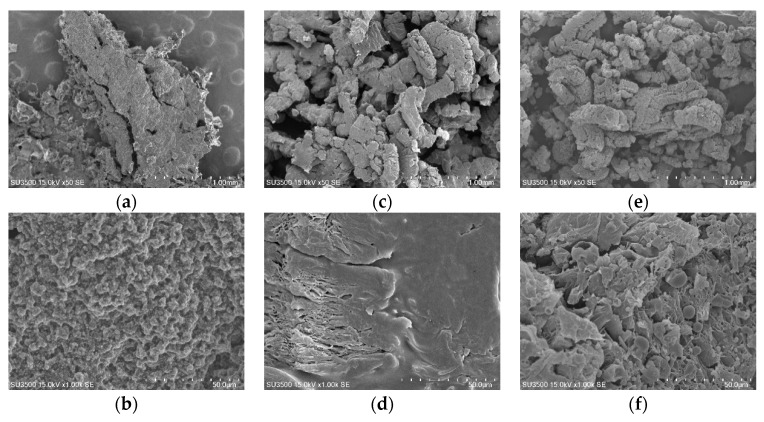
SEM images of the surface carbon residues for (**a**,**b**) EPDM/IFR, (**c**,**d**) EPDM/EG, and (**e**,**f**) EPDM/IFR/EG after the cone calorimeter test.

The digital photos and microstructures of the carbon residues after the cone calorimetric test are conducted with a digital camera and SEM in order to better understand the combustion behaviors of the EPDM materials, which are presented in Figure 7 and Figure 8, respectively. The flame retardant performance of the EPDM materials can be observed intuitively by the digital photos and microstructures of residues. EPDM will almost be completely burned, so only a very small amount of carbon residue can be observed for the pure EPDM. For the sample of EPDM/IFR, IFR can form a relatively dense carbon layer on the surface of the carbon residue, which can create a physical barrier toward the process of fire along the sample. Unfortunately, the carbon residue of EPDM/IFR has no obvious expansion and the carbon layer formed by IFR is discontinuous. In contrast, EPDM/EG has a high expansion ratio due to the extremely strong expansion capacity of EG, but the carbon layer of it is so weak that the expanded carbon residue is splashed out of the test mold. The SEM images show that a ‘‘worm-like’’ microstructure is developed for the carbon residue, which is very loose and leads to a weak carbon layer. When IFR and EG are added together, an integral, expanded, and compact carbon layer is formed, as shown in digital photographs, and a continuous dense microstructure is observed in the SEM images, which is mainly due to the interpenetration of the carbon residue of IFR and EG. The compact carbon layer has a good thermal insulation performance and can hinder the transfer of matter between the gas phase and the solid phase, improving the thermal insulation property, so as to delay and prevent the burning of materials. Therefore, the carbon layer formed by IFR and EG endows the EPDM materials with an excellent flame retardant property, which can decrease both mass and heat transfer and retard the degradation of the matrix, demonstrating a good synergetic effect of IFR and EG.

### 3.6. Possible Flame Retardant Mechanism

According to the above test results, the detailed flame retardant mechanisms of the flame retardant EPDM materials are concluded as follows. For EPDM/EG, EG expand and form a “worm-like” carbon layer to cover the matrix surface, which mainly manifested as solid phase flame retardant mechanism. For EPDM/IFR, in the gaseous phase, the decomposition of APP can generate ammonia so as to dilute the combustible gas. In the condensed phase, the decomposition of APP and PER can form chemical crosslinked bonds, resulting in a compact carbon layer, which can protect the inner matrix from swiftly decomposing. For EPDM/IFR/EG, IFR and EG have only physical interaction. When burning, the carbon residue will be expanded and maintains integrity. An interpenetrating and more compact carbon layer can be formed because of the char-forming ability of IFR and EG, and consequently endows the EPDM materials better flame ret ardant performances.

### 3.7. Combustion Process Analysis

**Figure 9:**
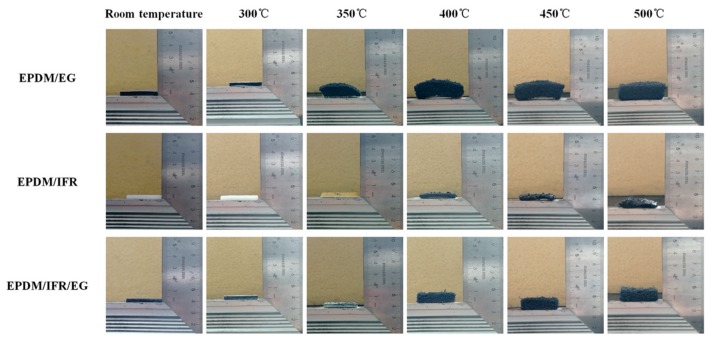
Digital photographs of the flame retardant EPDM materials after burning at different temperatures.

**Figure 10:**
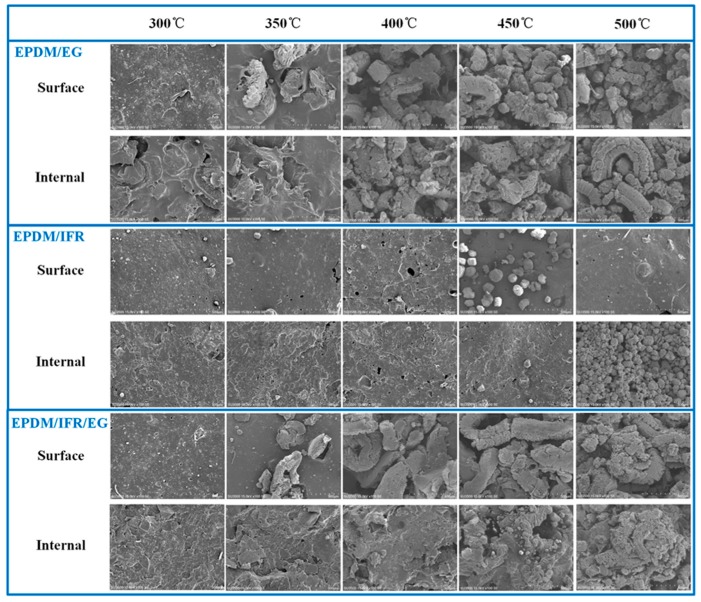
SEM images of the flame retardant EPDM materials after burning at different temperatures.

The results of the flame retardant tests show that IFR exhibits poor flame retardant effect for EPDM, and rather a high loading (60 phr) of flame retardant is required to achieve the necessary flame retardant performance. However, IFR shows a better flame retardant effect for many thermoplastics polymers, such as polypropylene, polyethylene, and ethylene–vinyl acetate copolymer [27,28,29]. In order to explain the reason of this phenomenon, the digital photos and microstructures of the flame retardant EPDM materials after burning in the muffle furnace at 300 °C, 350 °C, 400 °C, 450 °C, and 500 °C are conducted by digital camera and SEM to analyze the combustion process of the materials, which are presented in Figure 9 and Figure 10, respectively. The results show that the expansion ratio of EPDM/EG gradually increases as the temperature increases, and the carbon residue at 500 °C is six times higher than that of the unburned sample, demonstrating the extremely strong expansion capacity of EG. In contrast, the carbon residue of EPDM/IFR has no obvious expansion even if the temperature rises to 500 °C. The SEM images show that the surface of the carbon residue is compact, but unfortunately, the internal of the carbon residue is discontinuous. This phenomenon may be because EPDM will not melt during burning; as a result, APP and PER can only react partly and cannot form an expanded and continuous carbon layer. In contrast, the melting of these thermoplastic polymers during burning endues the overall material fluidity; therefore, the flame retardant particles are more likely to contact with each other, forming an expanded and continuous carbon layer. Consequently, IFR has a better flame retardant effect in these thermoplastics polymers than that of the EPDM materials. Meanwhile, as the expanded capacity of EG is much stronger than that of IFR, EPDM/EG shows better flame retardant property than EPDM/IFR. For EPDM/IFR/EG, due to both the expanded capacity of EG and char-forming ability of IFR, an expanded and more compact carbon layer is formed in the internal of the carbon residue. Although the degree of expansion is less than EPDM/EG, the more compact carbon layer and higher content of the carbon residue endows it with better flame retardant property.

## 4. Conclusions

The preparation of the intumescent flame retardant EPDM materials based on IFR and EG was demonstrated, in which the IFR and EG showed remarkable synergetic flame retardant effect on the EPDM materials. The resulting EPDM materials represent excellent flame retardant performances when the mass ratio of IFR and EG was 3:1 and the total addition content is 40 phr, which has a LOI value of 30.4% and a V-0 rating in the UL-94 vertical combustion test. Meanwhile, the heat release and smoke release of the EPDM composites are both decreased significantly in the cone calorimetry test. In addition, the synergetic flame retardant mechanism of APP/PER and EG is systematically investigated. The results show that IFR and EG have only physical interaction, and an expanded and compact carbon layer with a continuous dense microstructure is formed during combustion due to the complement of IFR and EG. Moreover, the result of combustion process analysis reveals that EPDM cannot melt during combustion, and its inability to form an expansion and continuous carbon layer may be the main cause of the poor flame retardancy of IFR in EPDM materials.

## Figures and Tables

**Table 1:** Formulation, limiting oxygen index (LOI) values, and UL-94 test results of the flame retardant ethylene–propylene–diene rubber (EPDM) materials. APP: ammonium polyphosphate, EG: expandable graphite, MA: melamine, PER: pentaerythrotol.

Sample	EPDM (phr)	APP (phr)	PER (phr)	MA (phr)	EG (phr)	Total Addition (phr)	LOI (%)	UL-94
1	100	-	-	-	-	-	17.8	NR
2	100	24	8	8	-	40	22.6	NR
3	100	36	12	12	-	60	26.2	V-0
4	100	30	10	-	-	40	26.2	V-0
5	100	45	15	-	-	60	32.2	V-0
6	100	-	-	-	30	30	24.4	V-0
7	100	-	-	-	40	40	26.0	V-0
8	100	-	-	-	50	50	26.4	V-0
9	100	26.2	8.8	-	5	40	29.2	V-0
10	100	22.5	7.5	-	10	40	30.4	V-0
11	100	18.8	6.2	-	15	40	29.6	V-0
12	100	11.2	3.8	-	5	20	26.2	V-0
13	100	16.8	5.7	-	7.5	30	28.2	V-0
14	100	28.1	9.4	-	12.5	50	31.8	V-0

**Table 2:** TGA parameters of the EPDM materials in the N_2_ atmosphere.

Sample	*T*_onset_ (°C)	*T*_max_ (°C)	d*W*/d*T* (%/min)	Residue (%)
EPDM	421.7	456.7	36.2	0
EPDM/IFR	239.1	458.7	19.0	8.0
EPDM/EG	300.5	451.9	16.2	24.0
EPDM/IFR/EG	259.9	460.1	19.3	12.4

**Table 3:** Burning parameters of the EPDM rubbers. TTI: time to ignition, PHRR: peak of heat release rate, THR: total heat release, TTPHRR: time to PHRR, FIGRA: the ratio of PHRR and TTPHRR, FRI: Flame Retardancy Index.

Sample	TTI (s)	PHRR (kW/m^2^)	THR (MJ/m^2^)	TTPHRR (s)	FIGRA (kW/(m^2^·s))	FRI	TSP (m^3^)	Residue (%)
EPDM	52	637	116.4	205	3.1	-	38.6	2.9
EPDM/IFR	78	346	84.9	160	2.2	3.79	25.1	21.3
EPDM/EG	35	408	80.0	155	2.6	1.53	14.2	25.7
EPDM/IFR/EG	70	197	77.6	115	1.7	6.52	16.3	43.9

**Table 4:** Element composition of carbon residues for the EPDM materials.

Sample	C (wt.%)	O (wt.%)	N (wt.%)	P (wt.%)
EPDM/EG	95.67	4.33	-	-
EPDM/IFR	34.62	47.49	6.13	11.76
EPDM/IFR/EG	59.70	30.80	3.44	6.06
